# A new record of the capped langur (*Trachypithecus pileatus*) in China

**DOI:** 10.24272/j.issn.2095-8137.2017.038

**Published:** 2017-07-18

**Authors:** Yi-Ming Hu, Zhi-Xin Zhou, Zhi-Wen Huang, Ming Li, Zhi-Gang Jiang, Jian-Pu Wu, Wu-Lin Liu, Kun Jin, Hui-Jian Hu

**Affiliations:** ^1^Guangdong Key Laboratory of Animal Conservation and Resource Utilization, Guangdong Public Laboratory of Wild Animal Conservation and Utilization, Guangdong Institute of Applied Biological Resources, Guangzhou Guangdong 510260, China; ^2^Institute of Zoology, Chinese Academy of Sciences, Beijing 100101, China; ^3^University of the Chinese Academy of Sciences, Beijing 100049, China; ^4^Forestry Inventory and Planning Institute of the Tibet Autonomous Region, Lhasa Tibet 850000, China; ^5^Research Institute of Forest Ecology Environment and Protection, Chinese Academy of Forestry, Beijing 100091, China

## DEAR EDITOR,

The distribution of the capped langur (*Trachypithecus pileatus*) in China has become controversial since Shortridge's langur (*Trachypithecus shortridgei*) was upgraded to a full species. The capped langur is considered to be distributed in northeast India, Bangladesh, Bhutan, and northwest Myanmar only (Brandon-Jones et al., 2004; Choudhury, 2008, 2014; Das et al., 2008; Groves, 2001). In our field survey, however, we obtained photos of the capped langur, demonstrating its existence in China.

Following the species promotion of Shortridge's langur (Brandon-Jones et al., 2004; Groves, 2001) and the delimiting of its distribution range to northwestern Yunnan in China and northeastern Myanmar (Brandon-Jones et al., 2004; Cui et al., 2016; Das et al., 2008; Groves, 2001; Htun et al., 2008), with a new record in southeastern Tibet (Wu et al., 2016), the capped langur has been deleted from the checklist of mammals in China (Jiang et al., 2015). Despite this, Dr. George Schaller has suggested that capped langurs might exist in the northeastern section of the Yarlung-Zangbo River (Choudhury, 2008).

**Figure 1:**
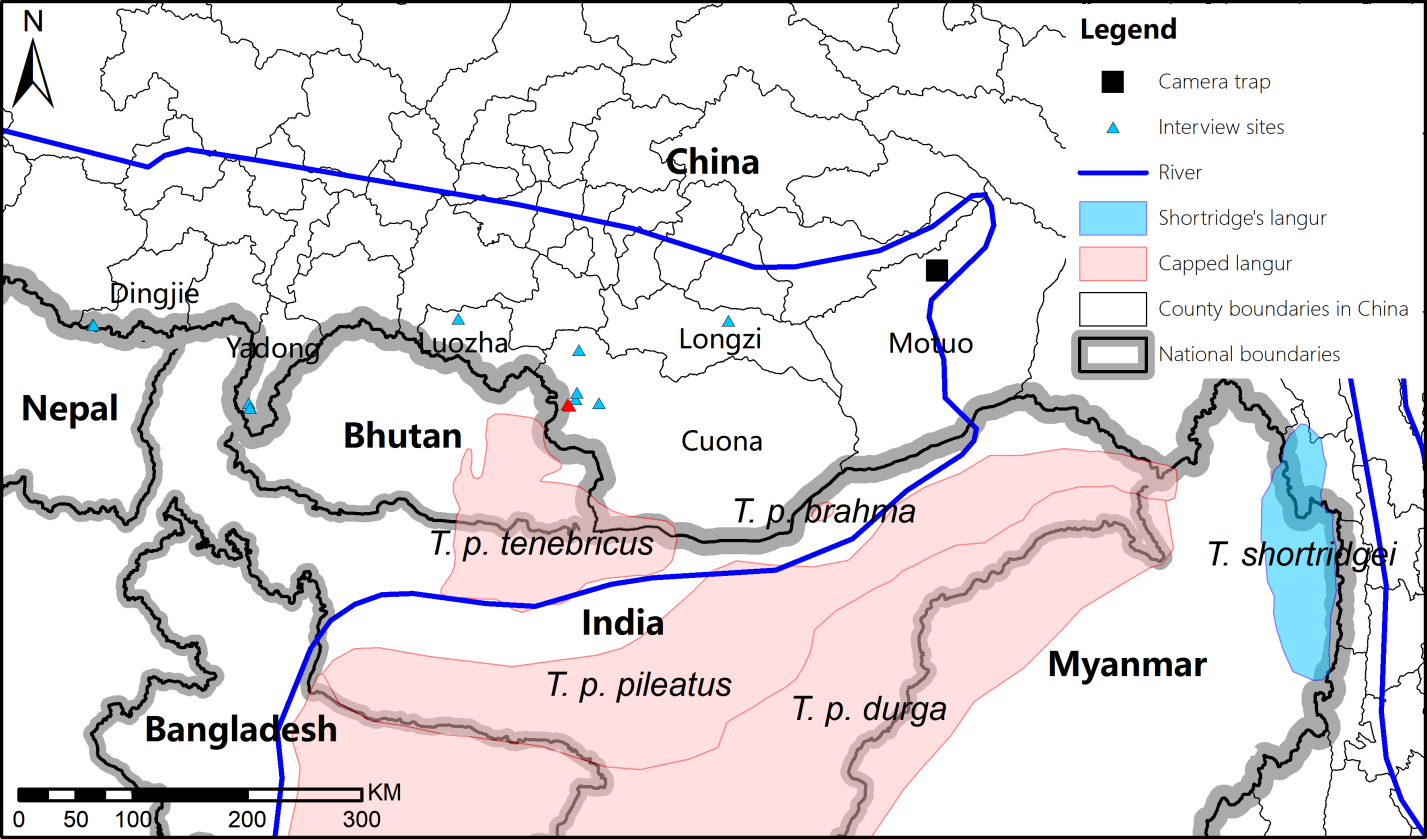
Map of langur survey area, including the counties within our study area

Recently, we conducted the Second National Survey of Terrestrial Wildlife Resources in southern Tibet along the southern slopes of the Himalayas in China (Figure 1) from 2013 to 2015 based on community interviews, field surveys (line transects), and camera traps in Dingjie, Yadong, Luozha, Cuona, Longzi, and Motuo counties. These counties encompass the potential distribution area of the langurs based on information from local forestry and conservation government departments in Tibet and previously published literature on langur species (Choudhury, 2008, 2014; Mittermeier et al., 2013; Smith & Xie, 2009; Wang, 2003). In this study, we aimed to: (1) determine if the capped langur exists in China, and (2) clarify its distribution in the southern Himalaya region.

Community interviews were conducted from June to August 2015. Following snowball sampling (Newing et al., 2011), we interviewed local leaders, forest patrollers, and regional forest managers. The interviewees were asked to describe the characteristics of the langur, and then identify the species from photos of several local langurs and macaques. The date, location, and group size of the langurs were recorded if the interviewees could correctly describe and identify the langur they encountered. We interviewed 41 people in 12 villages of the five surveyed counties, including 34 men and seven women. We conducted field surveys in habitats with low human disturbance near the villages where the community interview obtained positive feedback. We set line transects in subtropical and evergreen broad-leaved forest (suitable habitat for langur species) to corroborate the interview data. We set ten line transects in Cuona County, two in Dingjie County, and two in Yadong County from July to August 2015. The transects in Cuona County had a mean length of 8.6 km (range 2.8–15.7 km), with elevation ranging from 2 336 to 3 000 m a.s.l..The transects in the other two counties had a mean length of 28.9 km (range 7.3–97.3 km), with the elevation ranging from 2 100 to 3 000 m a.s.l.. The transect lines covered two types of woodland (evergreen broad-leaved forest, mixed broadleaf-conifer forest). We recorded all primate individuals and the latitude and longitude where they were found. To obtain valuable image information, we set 32 camera traps (Ltl 6210, Shenzhen Ltl Acorn Electronics Co. Ltd) in Gedang, Deyang Gully, and Xigong River of Motuo County from 16 October 2013 to 25 April 2014 (over 180 days). The camera traps were also placed in potential langur habitats (evergreen broad-leaved forest, mixed broadleaf-conifer forest).

**Figure 2:**
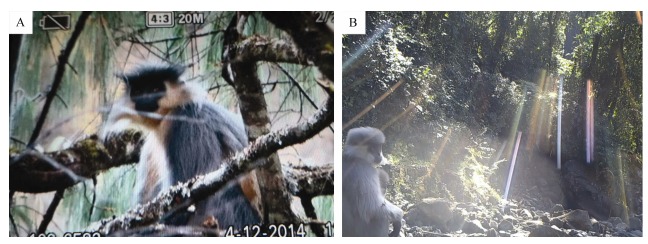
Photos of capped langur in Cuona County (A) and a langur species (possibly Shortridge's langur) in Motuo County (B)

One local person from Lai village and two from Xian village in Cuona County correctly described and identified the capped langur from other langurs and macaques, and also provided information that the capped langur population near the villages consisted of about 20–30 individuals (Table 1). Importantly, we obtained valuable photos of the capped langur subspecies *T. p. tenebricus* taken by local villagers in Lai village at noon (1200h) on 12 April 2014 (Figure 2A). Species identifications were consistent with the description of capped langurs in previous studies (Brandon-Jones et al., 2004; Choudhury, 2014; Groves, 2001). In the photos, at least four capped langur individuals (three adults and one infant) could be identified (Supplementary Figure S1 A, B). These photos provided strong evidence of the existence of the capped langur in Tibet, China. However, we did not find any capped langur individuals during the field survey. Previously, the distribution of the capped langur was considered to be northeast India, Bangladesh, Bhutan, and northwest Myanmar, with subspecies *T. p. tenebricus* distributed north of Brahmaputra River (lower part of the Yarlung-Zangbo River), including Bhutan and the Assam State of India, which neighbor Cuona County (Choudhury, 2008, 2014; Das et al., 2008). The habitat and topography in these regions provide the possibility of langur dispersal, with the lower range of the southern Himalayas a potential habitat for this species.

**1:** Details on the towns and villages in southern Tibet, China, where interviews were conducted between June and August 2015

Location	County	Number of Interviewees (*n*)
Chentang Town	Dingjie	4
Nadang Township	Dingjie	5
Chunpi Village	Yadong	3
Xia Yadong Township	Yadong	3
Zara Township	Luozha	3
Yumai Township	Luozha	3
Lampug Township	Cuona	3
Qudromo Township	Cuona	3
Marmang Township	Cuona	3
Lai Township	Cuona	4
Xian Village	Cuona	3
Gyiba Township	Cuona	1

Although no photo evidence was provided, a recent study indicated that Shortridge's langur is also distributed in southeastern Tibet (Wu et al., 2016). In the present study, a langur species (possibly Shortridge's langur) was twice captured by our camera trap (E94.90436°, N29.20175°, 1 429 m a.s.l.) in Deyang Gully, west of Xirang Township in Motuo County in January and March 2014 (Figure 2B and Supplementary Figure S2 A–C; three adults and an infant). However, as these pictures only caught the profiles of the species of interest, we cannot confirm with certainty that these are Shortridge's langurs. According to Choudhury (2014), the capped langur subspecies *T. p. brahma* is also distributed near Motuo County. Thus, the langur species we found in the Deyang Gully might also be *T. p. brahma* due to their similar gray coats. Further studies are needed to confirm the classification status of the langur species in Deyang Gully.

In our study, we confirmed the existence of the capped langur in China. As capped langurs are endangered, with small populations, the threat of habitat degradation and expanding human activities highlights the need for increasing conservation effort. Traditionally, Chinese mammalogists have used the Chinese name of “戴帽叶猴” for Shortridge's langur (*T. shortridgei*) (Jiang et al., 2015; Smith & Xie, 2009), which might cause confusion regarding the new record of capped langur (*T. pileatus*) in China. It is suggested that Shortridge's langur be named as “萧氏叶猴” and the capped langur be named as “戴帽叶猴”.

## ACKNOWLEDGEMENTS

We thank Wen-Wen Zhang and Ke Rong from the Northeast Forestry University, Wei-Shi Liu from the Chinese Academy of Forestry, and the local forestry administrations for assistance with the community interviews. We also thank the anonymous reviewers for their constructive suggestions for improving the quality of this manuscript.
